# Enhanced CO_2_ Adsorption on CeO_2_/SBA‐15: The Key Role of Oxygen Vacancies

**DOI:** 10.1002/cplu.202500288

**Published:** 2025-07-16

**Authors:** Danilo W. Losito, Jessica A. F. Pedro, Luís C. Cides‐da‐Silva, Matheus C. R. Miranda, Animesh Dutta, Rafael M. Santos, Tereza S. Martins

**Affiliations:** ^1^ Department of Chemistry Institute of Environmental, Chemical and Pharmaceutical Sciences Federal University of Sao Paulo Rua São Nicolau 210 Diadema 09913‐030 SP Brazil; ^2^ Institute of Physics Federal University of Sao Paulo Rua Do Matao 1371 Sao Paulo 05508‐090 SP Brazil; ^3^ Department of Civil, Environmental & Water Resources Engineering College of Engineering University of Guelph 50 Stone Rd E Guelph N1G 2W1 ON Canada

**Keywords:** CeO_2_, CO_2_ adsorption, mesoporous materials, oxygen vacancy, structure–activity relationships

## Abstract

This study investigates the role of oxygen vacancies in the CO_2_ adsorption and desorption dynamics of SBA‐15:CeO_2_ nanocomposites synthesized by direct (DS) and postsynthesis (PS) methods. Physicochemical analyses reveal that the DS method increases the concentration of oxygen vacancies and structural defects within the CeO_2_ framework, which significantly boosts CO_2_ adsorption capacity and strengthens the gas‐surface interactions. Among the materials, S_Ce4.a demonstrates the highest adsorption capacity, reaching 29.4 mg g^−^^1^ at 25 °C and 10.7 mg g^−1^ at 70 °C. These results indicate a physisorption mechanism governed by both thermal conditions and oxygen vacancies. Furthermore, S_Ce4.a and S_Ce10.a. exhibit remarkable stability over 20 adsorption–desorption cycles. The findings suggest that a lower cerium oxide content provides more accessible adsorption sites, making these materials promising candidates for high‐performance. Overall, this work highlights synergistic interplay between oxygen vacancies and mesoporous structures, paving the way for the rational design of advanced materials for CO_2_ capture technologies.

## Graphical abstract

Mesostructured nanocomposites (SBA‐15:CeO_2_), synthesized via two different methods, are analyzed to highlight their structural and morphological differences. The samples are tested for CO_2_ adsorption/desorption at 25, 35, 50, and 70 °C, with enhanced adsorption capacity attributed to increased oxygen vacancies induced by the direct synthesis method.

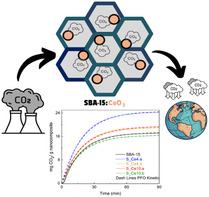

## 1 Introduction

Since the Industrial Revolution, greenhouse gases (GHGs) have significantly disrupted the Earth's climate system, increasing the temperature of the planet. The concentration of GHGs in the atmosphere is reinforced by population growth and the consequent demand for modern lifestyles, leading to intensified fossil fuel consumption. As a result, the atmospheric levels of methane (CH_4_), nitrous oxide (N_2_O), and carbon dioxide (CO_2_) have risen to 262%, 126%, and 149% of pre‐industrial levels by 2020, respectively.^[^ ^1^, ^2^ ^]^ In 2015, 191 nations convened at the Paris Agreement during the United Nations Climate Conference to set measures aimed at limiting global temperature increases to 1.5 °C, or at most 2 °C, above preindustrial levels.^[^ ^3^ ^]^ Therefore, to achieve these goals and avoid alarming climate scenarios, it is imperative to rapidly reduce GHG emissions. Among the strategies explored, carbon capture technologies have emerged as a crucial focus for mitigating CO_2_ emissions.^[^ ^4^, ^5^, ^6^ ^]^

These technologies include Carbon Capture Storage (CCS)^[^ ^7^, ^8^, ^9^ ^]^ and Carbon Capture Utilization (CCU),^[^ ^10^, ^11^, ^12^ ^]^ which leverage advanced materials such as zeolites,^[^ ^13^, ^14^, ^15^ ^]^ MOF's,^[^ ^16^ ^]^ biochar,^[^ ^17^, ^18^ ^]^ clay minerals,^[^ ^19^, ^20^, ^21^, ^22^ ^]^ and mesoporous silica.^[^ ^23^, ^24^, ^25^, ^26^ ^]^ Many of these adsorbents are reported grafted with amino group and presenting high adsorption capacity.

Despite their potential, amine‐based solid sorbents face significant challenges due to their corrosive nature, high cost, elevated regeneration energy requirements, and low thermal degradation temperature, all of which limit their commercial viability, as highlighted in two recent reviews articles (Lashaki et al.^[^ ^27^ ^]^ and Zhao et al.^[^ ^28^ ^]^). Despite their promise, certain adsorbents involve hazardous compounds and costly preparation methods, limiting their commercial viability. Consequently, ongoing research is essential to develop stable and sustainable solid adsorbents with optimized physicochemical properties for enhanced CO_2_ capture.^[^ ^25^, ^29^, ^30^, ^31^, ^32^ ^]^

For instance, Kim et al.^[^ ^18^ ^]^ and Singh et al.^[^ ^33^ ^]^ investigated the CO_2_ adsorption capacity of amino‐free sorbents, focusing on the development of sustainable materials with improved properties for long‐term application. More recently, Mahendran et al.^[^ ^34^ ^]^ studied SBA‐15 under various experimental conditions, including feed flow rate, adsorption temperature, CO_2_ concentration, and adsorbent loading. At 30 °C, SBA‐15 exhibited a CO_2_ adsorption capacity of 6.43 mg g^−^^1^. The study highlights the material's facile regeneration, enabling multiple adsorption–desorption cycles without significant loss in capacity.

Regarding the physical properties of adsorbents, Oliveira et al.^[^ ^35^ ^]^ compared two types of ordered mesoporous silica (MCM‐41 and SBA‐15) which exhibit distinct structural, textural, and morphological characteristics. These materials were impregnated with varying amounts of chitosan and evaluated for CO_2_ adsorption at 25 °C and 1 bar, achieving capacities ranging from 22 to 35 mg g^−^^1^. The results underscore the importance of the pore network in impregnated materials, as it can influence the accessibility and distribution of adsorption sites within the nanocomposite.

Ordered mesoporous silica (OMS), such as SBA‐15, MCM‐41, and FDU‐12, exhibit interesting properties that can be enhanced through various synthesis techniques and the impregnation of diverse organic and inorganic species. These modifications significantly enhance their potential for applications, particularly in the adsorption of pollutant gases like CO_2_, making them highly attractive for environmental and industrial purposes.^[^ ^34^, ^35^ ^]^ Among OMS, SBA‐15 stands out for its unique structural and functional properties, including uniform and tunable pores (2–50 nm), high surface area (800 m^2^ g^−1^), abundant silanol groups (Si‐OH), and high thermal and mechanical stability.^[^ ^36^, ^37^, ^38^ ^]^ The silanol group allows SBA‐15 to impregnate some species, such as metal oxide nanoparticles and biological molecules.^[^ ^39^, ^40^, ^41^, ^42^ ^]^ While numerous studies have explored the functionalization of SBA‐15 with amino groups and other molecules to enhance CO_2_ adsorption,^[^ ^41^, ^43^, ^44^ ^]^ far fewer have focused on its modification with inorganic species like metal oxides. This approach holds significant promise, as metal oxides offer the unique ability to alter structural properties and introduce defects that can amplify CO_2_ adsorption capacity.

CeO_2_ stands out among inorganic materials due to its unique structural and redox characteristics, stemming from the Ce^3^^+^/Ce^4^^+^ pair. Enhancing the proportion of Ce^3^^+^ within its structure promotes the formation of oxygen vacancies (O.V.), a pivotal attribute that strengthens its ability to interact with CO_2_ in both adsorption and catalytic applications.^[^ ^45^, ^46^, ^47^, ^48^, ^49^, ^50^, ^51^, ^52^, ^53^ ^]^ Although this property is well reported in the literature for catalysis of CO_2_, there is a relative lack of information regarding the use of CeO_2_ and the influence of O.V. on the CO_2_ adsorption process.

This study focuses on the development of SBA‐15:CeO_2_ nanocomposites with varying ceria molar proportion (4 and 10 mol%), synthesized using postsynthesis (PS) and direct synthesis (DS) methods. These materials are evaluated for their structural, textural, and morphological properties, as well as their CO_2_ adsorption–desorption behavior at different temperatures (25, 35, 50, and 70 °C). The adsorption kinetics are analyzed using the pseudo‐first‐order (PFO) model. In addition, cyclic stability tests were conducted over 20 adsorption–desorption cycles at 35 °C for the materials prepared by DS method.

This study presents a novel strategy for synthesizing SBA‐15:CeO_2_ nanocomposites with tailored oxygen vacancies, aiming to enhance long‐term CO_2_ capture performance. By exploring the impact of synthesis methods on the structural and morphological properties, it addresses key gaps in the design of mesoporous composite adsorbents.

## 2 Experimental Section

### 2.1 Materials and Chemicals

Tetraethyl orthosilicate (TEOS, 98%, Sigma–Aldrich, Brazil), nonionic triblock copolymer surfactant PluronicP123 (poly(ethylene oxide)‐poly(propylene oxide)‐poly(ethylene oxide), EO_2__0_PO_70_EO_2__0_, Sigma–Aldrich, Brazil), hydrochloric acid (HCl, 37%, Synth, Brazil), cerium(III) nitrate hexahydrate (99.99%, Sigma–Aldrich, Brazil), nickel(III) nitrate hexahydrate (99.99%, Sigma–Aldrich, Brazil), and ethanol (EtOH, Sigma–Aldrich, Brazil).

### 2.2 Synthesis of the Ordered Mesoporous Material SBA‐15

SBA‐15 was synthesized following the methodology described by Zhao et al.^[^ ^36^ ^]^ with minor modifications, as follows: 4.0 g of Pluronic P123 were dissolved in 120 mL of HCl (2 mol L^−1^) and 30 mL of deionized water under stirring for 1 h at room temperature. Subsequently, 8.9 mL of TEOS was added, and the mixture was stirred for 24 h at 40 °C and hydrothermally aged for 48 h at 100 °C in an autoclave. The resulting material was filtered, washed thoroughly with deionized water to remove chloride ions, and dried at 60 °C. The template (Pluronic P123) was removed by solvent extraction using ethanol, following by calcination under air atmosphere at 540 °C, using a heating rate of 1 °C min^−1^ and an air atmosphere. Upon reaching this temperature, it was maintained under an air atmosphere for 3 h to eliminate the carbonaceous material.

### 2.3 Synthesis of the SBA‐15:CeO_2_ Nanocomposites

The synthesis of S_Ce10 and S_Ce4 nanocomposites was performed by two methods: direct synthesis and postsynthesis, as described in the following.

#### 2.3.1 Direct Synthesis

2.0 g of Pluronic P123 was dissolved in 75 mL of HCl (1.6 mol L^−1^), followed by the addition of 10% or 4% (molar ratio Ce/SiO_2_) of Ce(NO_3_)·3.6H_2_O presolubilized in 20 mL of EtOH. Afterward, 4.45 mL of TEOS was added, and the mixture was stirred for 24 h at 40 °C and hydrothermally aged for 48 h at 100 °C. The resulting material was stirred at 80 °C until complete solvent evaporation and then calcinated at 540 °C, using a heating rate of 1 °C min^−1^ in an air atmosphere. This temperature was maintained for 3 h to eliminate the carbonaceous material.

#### 2.3.2 Postsynthesis

The synthesized SBA‐15 was dispersed in 20 mL of EtOH, followed by the addition of 10% or 4% (molar ratio Ce/SiO_2_) of the metal precursor Ce(NO_3_)·3.6H_2_O presolubilized in 20 mL of EtOH. The mixture was stirred for 24 h at room temperature, followed by stirring at 80 °C until complete solvent evaporation. Then, the material was calcined at 540 °C with heating rate of 1 °C·min^−1^ in air. This temperature was maintained for 3 h to eliminate the carbonaceous material. **Table** **1** presents the nomenclature of the materials studied in this article and the amount of each metal precursor to prepare the nanocomposites.

**Table 1:** Materials prepared in this work. Note that the suffixes “a” and “b” indicate the synthetic method used: direct synthesis and postsynthesis, respectively. The proportion presented considers the molar ratio of Ce to SiO_2_ (nCe/nSiO_2_).

Name	Description + molar ratio n_Ce_/n_SiO2_
SBA‐15	Pure SBA‐15
S_Ce10.a	SBA‐15 + 10% Ce/SiO_2_ (direct synthesis)
S_Ce10.b	SBA‐15 + 10% Ce/SiO_2_ (postsynthesis)
S_Ce4.a	SBA‐15 + 4% Ce/SiO_2_ (direct synthesis)
S_Ce4.b	SBA‐15 + 4% Ce/SiO_2_ (postsynthesis)

### 2.4 Characterization Techniques

Small‐angle X‐ray scattering (SAXS) curves were acquired on a Nanostar (Bruker) instrument, with a point beam generated by a conventional copper tube (CuK_α_ = 0.15418 nm), a current of 30 mA, and an accelerated tension of 40 kV. The setup utilized Göbel mirror geometry, a bi‐dimensional detector, with 64.8 cm (0.2 ≤ *q* ≤ 3.5 nm^−1^) between the sample and detector.

X‐ray diffraction (XRD) measurements were performed using a Rigaku Ultima + diffractometer with CuK_α_ radiation (*λ* = 0.15418 nm) employing the powder method. The selected angular range was from 5° to 90° (2*θ*), with a step size of 0.05°, a counting time of 2.0 s, and operating at 40 kV and 30 mA, with sample support rotating at 30 rpm.

X‐ray photoelectron spectra (XPS) were obtained at LNNano‐CNPEM, Brazil, using a Thermo Scientific K‐Alpha X‐ray Photoelectron Spectrometer System using a monochromatic Al K‐Alpha (1486.6 eV) source. The analysis was performed with 10 scans with a spot size of 300 μm, a pass energy of 50.0 eV, an energy step size of 0.10 eV, and a dwell time of 50 ms. The acquired data were analyzed using the Casa XPS software (version 2.1.0.1). For the Ce3d spectra, the peak deconvolution was performed using the Gaussian method with Origin software.

Raman spectroscopy data were obtained using a Renishaw InVia Raman microscope equipped with a CCD multichannel detector, He‐Ne lasers (632.8 nm), and diode lasers (830 nm). The spectra were acquired with a spectral resolution of 4 cm^−1^, using three accumulations, an integration time of 20 s, and a spectral range from 100 to 2000 cm^−1^.

Fourier transform infrared (FTIR) spectra were recorded in the range 4000−400 cm^−1^, using an Agilent Cary 630 FTIR spectrometer. IR measurements were performed in attenuated total reflection (ATR) mode.

Nitrogen adsorption/desorption isotherms (NAI) were recorded on NOVA 2 porosimeter (Micromeritics ‐ ASAP 2020) after degassing at 200 °C (until vacuum ≤ 10 μm Hg) for ≈12 h. The specific surface areas of mesoporous and microporous were calculated through BET method^[^ ^54^ ^]^ (Brunauer–Emmet–Teller) and the t method^[^ ^55^ ^]^ (“t‐plot”), respectively. Pore size distribution and cumulative pore volume were determined using the BJH method^[^ ^56^ ^]^ (Barrett–Joyner–Halenda) of adsorption.

Scanning electron microscopy (SEM) images were acquired using a JSM‐6610LV (JEOL) instrument, operating with a secondary electron imaging (SEI) detector. Prior to measurement, samples were placed onto conductive double‐sided adhesive carbon tape and coated with a thin layer of gold.

Transmission electron microscopy (TEM) images were acquired using the JEOL JEM‐2100 F. The equipment operated at a voltage of 200 kV. For the sample preparation, a drop was deposited in a copper micro‐net coated with carbon tape.

Thermogravimetric analysis (TGA) and differential scanning calorimetry (DSC) measurements were conducted using a TA Instruments Discovery SDT 650 Simultaneous Thermal Analyzer DSC/TGA system. The data were obtained at a heating rate of 10 °C min^−1^, within a temperature range from 35 to 1000 °C, under a dynamic air atmosphere (100 mL min^−1^), employing an alumina crucible with ≈5 mg of the sample.

### 2.5 CO_2_ Adsorption Tests

The CO_2_ adsorption test was conducted following a systematic procedure, using an SDT Q600 of TA Instruments. Initially, 7 mg of each sample was submitted to pretreatment at 200 °C for 1 h under a nitrogen flow (70 mL min^−1^) to eliminate adsorbed water and impurities. Subsequently, the temperature was lowered to the desired value, maintaining the nitrogen flow to achieve mass equilibrium. Once stabilized, the nitrogen gas was replaced with CO_2_, and the flow rate was increased to 90 mL min^−1^. The system was then exposed to CO_2_ for 90 min to measure the adsorption capacity of the nanocomposites. Following this step, the flow was switched back to nitrogen at 70 mL min^−1^ for a 60 min desorption phase. All samples were tested at 35 °C, while the standout nanocomposite samples, S_Ce4.a and S_Ce10.a, were additionally tested at 25, 50, and 70 °C to further evaluate their performance across a broader temperature range. This variation in temperature provided valuable insights into the kinetics of adsorption, highlighting the influence of temperature on adsorption performance. The stability of the S_Ce4.a and S_Ce10.a was performed for 20 cycles at 35 °C similarly to the description in CO_2_ adsorption test with the unique difference in the time of desorption which was kept for 90 min.

## 3 Results and Discussion

**Figure 1:**
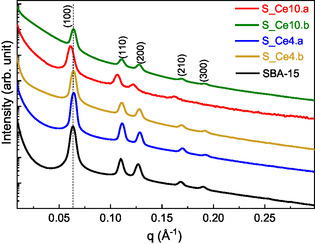
SAXS measurements of all nanocomposites prepared: SBA‐15, nanocomposites containing 4% of Ce synthesized by direct synthesis method (S_Ce4a, S_4Ceb), and nanocomposites containing 10% of Ce synthesized by postsynthesis method (S_Ce10a, S_Ce10b).

The SAXS results for SBA and the nanocomposites prepared by DS and PS are displayed in **Figure** **1** and **Table** **2**. The curves exhibited five typical peaks of the SBA‐15 ordered mesoporous structure, which were indexed as (100), (110), (200), (210), and (300) reflections. These peaks confirm that both methods of synthesis do not collapse the 2D hexagonal structure with space group *p6mm*, characteristic of the ordered mesoporous silica type SBA‐15.^[^ ^57^, ^58^ ^]^

**Table 2:** Structural properties determined from SAXS measurements for S_IL and S_IL:Ce samples.

Samples	*d*_(hkl)_ a)/*a*_(hkl)_ b) [nm]				
	(100)	(110)	(200)	(210)	(300)
SBA	9.9/11.4	5.7/11.4	5.0/11.4	3.7/11.4	3.3/11.4
S_Ce10.a	10.4/12.0	5.9/11.7	5.1/11.9	3.9/11.8	–
S_Ce10.b	9.8/11.4	5.7/11.4	4.9/11.3	3.7/11.4	3.3/11.4
S_Ce4.a	9.8/11.3	5.6/11.3	4.9/11.3	3.7/11.3	3.3/11.3
S_Ce4.b	9.8/11.4	5.7/11.3	4.9/11.3	3.7/11.3	3.3/11.4

a) *d*_(hkl)_ = interplanar distance.

b) *a*_(hkl)_ = lattice parameter. The error of the lattice parameter is 2%.

The area under the (100) peak was analyzed for all prepared materials using the Gaussian fitting method in Origin software, providing valuable insights into the incorporation of CeO_2_ within the SBA‐15 framework. These values are presented in Table S1, Supporting Information. Comparing the results, a decrease in the (100) peak area is observed for all nanocomposites when compared to pure SBA‐15. This reduction is attributed to the impregnation of inorganic metal species within the mesoporous channels of the matrix.^[^ ^58^, ^59^ ^]^ Notably, the materials prepared using the PS method exhibit a more pronounced decrease, indicating a higher loading of nanoparticles integrated into the mesostructure compared to those prepared using the DS method.

Additionally, slight shifts to lower values of q (Å^−^^1^) were observed for the DS nanocomposite containing 10% Ce nanoparticles (S_Ce10.a), which are attributed to increased interplanar distances (*d*_hki_) and lattice parameters (*a*_hki_). This phenomenon can be explained by the salting‐in effect, where the nitrate precursors used during the synthesis of SBA‐15 nanocomposites interact with the structure‐directing agent, leading to an expansion of the mesoporous structure.^[^ ^60^, ^61^ ^]^ This effect is not observed for the sample containing 4% due to the small amount of nitrate precursor in the synthesis. The values of *d*_(hkl)_ and *a*_(hkl)_ for all materials prepared are shown in Table 2.

**Figure 2:**
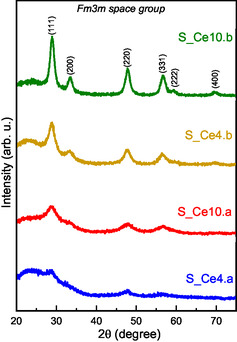
XRD results for the samples S_Ce4.a, S_Ce4.b, S_Ce10.a, and S_Ce10.b.

The XRD curves for the SBA‐15:CeO_2_ nanocomposites are displayed in **Figure** **2**. All curves exhibit a broad peak in 22.5° (2*θ*°), attributed to the amorphous silica characteristic of SBA‐15. Additionally, the data reveal the principal reflections corresponding to the fluorite structure of CeO_2_ (space group of *Fm‐3m)*. These reflections are observed at 2*θ*° values of 29.0°, 33.4°, 47.9°, 56.6°, 59.4°, 69.7°, and 76.8°, indexed as the (111), (200), (220), (311), (222), (400), and (331) Miller indices, respectively.^[^ ^62^, ^63^ ^]^

Comparing the materials prepared by direct synthesis and postsynthesis, the DS nanocomposites exhibit lower intensity and broader CeO_2_ diffraction peaks. This suggests that the impregnation method of the cerium precursor within the SBA‐15 mesoporous structure influences the crystallization process of CeO_2_. Consequently, the DS nanocomposites show cerium nanoparticles with lower crystallinity, yielding crystallite sizes of 4.2 nm for S_Ce10.a and 4.1 nm for S_Ce4.a. In contrast, the PS nanocomposites display sharper and more intense CeO_2_ reflections indicating higher crystallinity and larger crystallite sizes of 9.1 nm for S_Ce10.b and 6.4 nm for S_Ce4.b. The crystallite sizes were calculated using the Scherrer equation (S1), and the resulting values are summarized in **Table** **3**.

**Table 3:** Crystallite size (*d*_(hkl)_) of SBA‐15:CeO_2_ samples (S_Ce4.a, S_Ce10.a, S_Ce4.b, S_Ce10.b), calculated using the Scherrer equation based on the peaks (111) and (220) diffraction peaks.

Sample	Crystallite size *d*_(hkl)_ Ce [nm]
S_Ce4.a	4.1
S_Ce10.a	4.2
S_Ce4.b	6.7
S_Ce10.b	9.1

**Figure 3:**
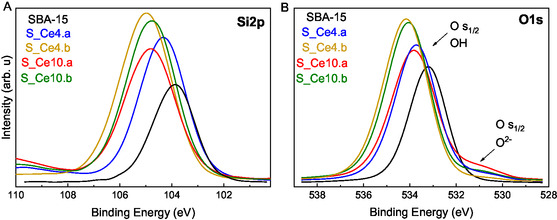
XPS spectra A) Si2p and B) O1s of the SBA‐15 and its nanocomposites containing Ce: S_Ce4.a, S_Ce4.b, S_Ce10.a, and S_Ce10.b.

**Figure** **3**A,B displays XPS spectra data of SBA‐15 pure and the cerium nanocomposites for Si2p and O1s analysis, respectively. The Si2p_3/2_ peak is observed within the binding energy range of 102–108 eV, corresponding to silicon in silanol (Si—OH) and siloxane (Si—O—Si) groups within the silica framework. For SBA‐15, this peak appears at 103.8 eV, whereas for the nanocomposites, a shift toward higher binding energies is observed. This shift is attributed to the electrostatic interaction between CeO_2_ nanoparticles and the SBA‐15 support via Si—OH groups.^[^ ^64^ ^]^

The O1s spectra reveal a prominent peak between 533 and 535 eV, which is attributed to the oxygen in Si—OH and Si—O—Si bonds, as well as adsorbed water within the mesoporous structure.^[^ ^64^, ^65^ ^]^ Additionally, a minor band is observed between 530 and 532 eV for the nanocomposites, corresponding to the O^2^^−^ lattice species of the CeO_2_ structure and surface‐adsorbed oxygen, both of which are associated with the presence of oxygen vacancies.^[^ ^65^, ^66^, ^67^ ^]^ Notably, the nanocomposites synthesized via the DS method exhibit a more intense signal for this minor band, suggesting that the synthesis method influences the CeO_2_ structure formation, likely due to interactions between the cerium precursor and the reactants during the preparation of the ordered mesoporous silica.

**Figure 4:**
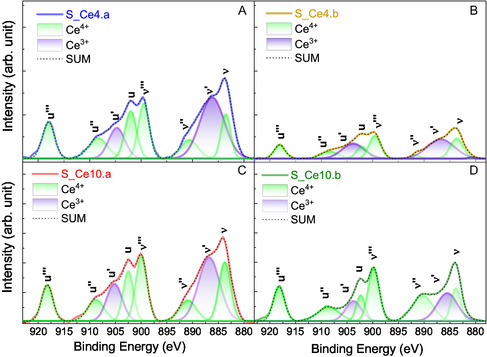
XPS spectra of Ce3d for the nanocomposites: A) S_Ce4.a, B) S_Ce4.b, C) S_Ce10.a, and D) S_Ce10.b. The green peaks are assigned to Ce^4+^ and the purple to Ce^3+^.

These findings are further supported by Ce3d spectra, presented in **Figure** **4**A–D. All nanocomposites exhibit characteristic peaks of ceria, indicating the presence of both Ce^4^^+^ and Ce^3^^+^ ions. The Ce3d_5/2_ peaks, observed at 882.1, 884.1, 888.4, and 897.5 eV, are labeled as v, v′, v″, and v″, respectively, in the spectra. For the Ce3d_3/2_ region, peaks at 901, 904, 908, and 919.6 eV are labeled as u, u′, v″, and v″.^[^ ^45^, ^47^, ^48^, ^65^, ^66^, ^67^, ^68^, ^69^, ^70^, ^71^, ^72^, ^73^ ^]^ Peaks associated with Ce^4^^+^ (v″′, u″, u, v″′, v″, and v) are indicated in green, while those corresponding to Ce^3^^+^ ions (u′ and v′) are highlighted in purple. Deconvolution of these Ce3d peaks allows for the determination of the oxygen vacancy concentration by calculating the ratio of the integrated areas corresponding to each oxidation state (Ce^3^^+^/Ce^4^^+^). The resulting ratios, summarized in **Table** **4**, reveal a greater contribution of the Ce^3^^+^ across the samples in the following order: S_Ce4.a > S_Ce10.a > S_Ce4.b > S_Ce10.b. These results underscore that both synthesis methods effectively generate oxygen vacancies within the CeO_2_ structure. However, the synthesis method plays a crucial role in influencing the oxygen vacancy concentration, with the direct synthesis method resulting in a higher concentration of oxygen vacancies compared to the postsynthesis method.

**Table 4:** Oxygen vacancy percentage of SBA‐15:CeO_2_ nanocomposites: S_Ce4.a, S_Ce10.a, S_Ce4.b, S_Ce10.b. The values were calculated through the ratio of the corresponding peak area of Ce^4+^ and Ce^3+^.

Sample	Ce^3+^/ (Ce^4+^/Ce^3+^) [%]
S_Ce4.a	43
S_Ce10.a	39
S_Ce4.b	35
S_Ce10.b	27

**Figure 5:**
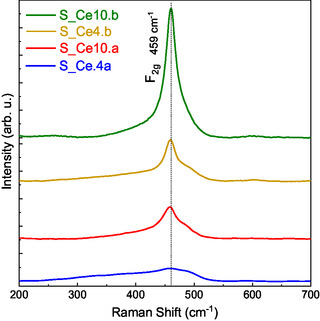
Raman spectra for the SBA‐15:CeO_2_ nanocomposites S_Ce4a, S_Ce4b, S_Ce10a, and S_Ce10 b.

**Figure** **5** presents the Raman spectra for the SBA‐15:CeO_2_ nanocomposites. The signal at 460 cm^−^^1^ corresponds to the F2g vibrational mode, characteristic of the cubic fluorite structure of CeO_2_, consistent with the findings from the XRD analysis. Additionally, a slight shift to lower Raman wavelengths, along with a broadened F2g peak, is observed and attributed to structural defects that disrupt the Ce–O symmetry.^[^ ^74^, ^75^ ^]^ The presence of these defects becomes more pronounced in the following order: S_Ce4.a < S_Ce10.a < S_Ce4.b < S_Ce10.b, suggesting that the direct synthesis method induces a higher concentration of structural defects in the CeO_2_ nanoparticles. This observation is likely due to stronger interactions between the cerium precursors and the silica framework during the direct synthesis process.^[^ ^47^, ^75^, ^76^ ^]^ Furthermore, a lower cerium content appears to result in more pronounced structural defects compared to samples with higher cerium content. These observations are consistent with the data obtained from XRD and XPS analyses.

The FTIR spectra of SBA‐15 and the nanocomposites containing CeO_2_ are shown in Figure S1, Supporting Information. All materials exhibit the characteristic bands of the SBA‐15 structure. The band around 1635 cm^−1^ corresponds to H—O—H bending vibration of adsorbed H_2_O. Bands at ≈1055, 965, 800, and 440 cm^−1^ are assigned to the asymmetric stretching vibration of Si—O—Si, the stretching vibration of Si—OH, the symmetric stretching vibration of Si—O—Si, and the bending vibration of Si—O—Si, respectively.^[^ ^59^, ^63^, ^77^ ^]^

TGA curves for the prepared materials are shown in Figure S2A,B (SBA‐15, S_Ce.a and S_Ce.b), Supporting Information. For all samples, the initial weight loss observed in the temperature range of 30–120 °C is attributed to the elimination of physically adsorbed water from the material's surface. The second weight loss event, occurring between 120 and 1000 °C, corresponds to the condensation of silanol groups on the silica surface. Pure SBA‐15 exhibits a 7.4% weight loss in the first event and 2.5% in the second event, consistent with previous studies reported by our group.^[^ ^59^ ^]^

Materials prepared via the direct synthesis method show higher amounts of adsorbed water compared to those synthesized postsynthesis method. Specifically, S_Ce4.a and S_Ce10.a exhibit weight losses of 13.5% and 8.2%, respectively, in the first event, while S_Ce4.b and S_Ce10.b demonstrate lower weight losses of 11.2% and 6.3% in the same temperature range. Moreover, samples containing 4% cerium exhibit a higher amount of eliminated water due to increased free volume within these samples, resulting in lower residual mass values of 82.9% for S_Ce4. and 85.9% for S_Ce4.b at the end of experiment. Table S2, Supporting Information, summarizes the weight change and onset temperature (T_onset_) values for each event, as well as the final residue.

**Figure 6:**
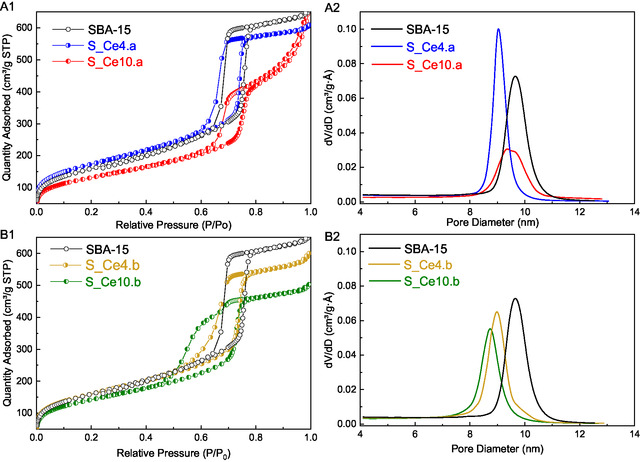
A1,B1) N_2_ adsorption–desorption isotherms and A2,B2) pore size distributions for the prepared materials: SBA‐15, S_Ce10.a, S_Ce10.b, S_Ce4.a, and S_Ce4.b.

**Figure** **6**A,B displays the N_2_ physisorption isotherms and the pore size distribution obtained using the BJH method for the SBA‐15 and the SBA‐15:CeO_2_ nanocomposites. All materials exhibit type IV physisorption isotherms with H1 hysteresis loops (Figure 6A1,B1), in accordance with the International Union of Pure and Applied Chemistry (IUPAC) classification.^[^ ^78^, ^79^ ^]^ These results are characteristic of hexagonal bidimensional mesoporous materials, such as SBA‐15. The pore size distribution for pure silica is ≈10 nm, aligning with values reported in the literature.^[^ ^80^, ^81^ ^]^

Comparing the results of SBA‐15:CeO_2_ nanocomposites with pure SBA‐15, a decrease in nitrogen adsorption is observed. This decrease is attributed to the presence of cerium oxide species within the mesoporous channels of SBA‐15, becoming more pronounced with increase cerium oxide loading. The pore size distributions for the SBA‐15:CeO_2_ nanocomposites (Figure 6A2,B2) indicate a smaller average pore diameter for samples prepared via the PS method, measuring 8.7 nm for S_Ce10.b and 8.9 nm for S_Ce4.b. In comparison, the DS nanocomposites exhibit slightly larger and broader pore size distributions, with average values of 9.5 nm for S_Ce10.a and 9.1 nm for S_Ce4.a. Additionally, the sample containing 4% CeO_2_ shows a narrower pore size distribution than the sample with 10% CeO_2_. Analysis of the pore volume (V_p_) of the materials reveals that the direct synthesis method results in a higher value compared to postsynthesis. These findings are consistent with data obtained from XRD, SAXS, SEM, and TEM, underscoring that the volume occupied by nanoparticles within the mesoporous channels of SBA‐15 is greater for PS nanocomposites. **Table** **5** summarizes the textural properties, including specific surface area (S_BET_), pore size (D_BJH_), and pore volume (V_p_), derived from N_2_ adsorption isotherms.

**Table 5:** Specific surface area (*S*_BET_), pore size distribution (*D*_(BJH)_), pore volume (Vp), oxygen vacancy (O.V.), and CO_2_ adsorption for pure SBA‐15 and SBA‐15:CeO_2_ nanocomposites.

Samples	N_2_ sorption isotherms				
	S_(BET)_ a) [m^2^ g^−1^]	D_(BJH)_ b) [nm]	V_p_ c) [cm^3 ^g^−1^]	O.V.d) [%]	Adsorption of CO_2_ [mg g^−1^]
SBA‐15	573	9.6	1.38	–	17.1
S_Ce10.a	471	9.5	1.28	39	19.3
S_Ce10.b	510	8.7	1.10	27	16.3
S_Ce4.a	621	9.1	1.34	43	24.3
S_Ce4.b	573	8.9	1.09	35	18.6

**Figure 7:**
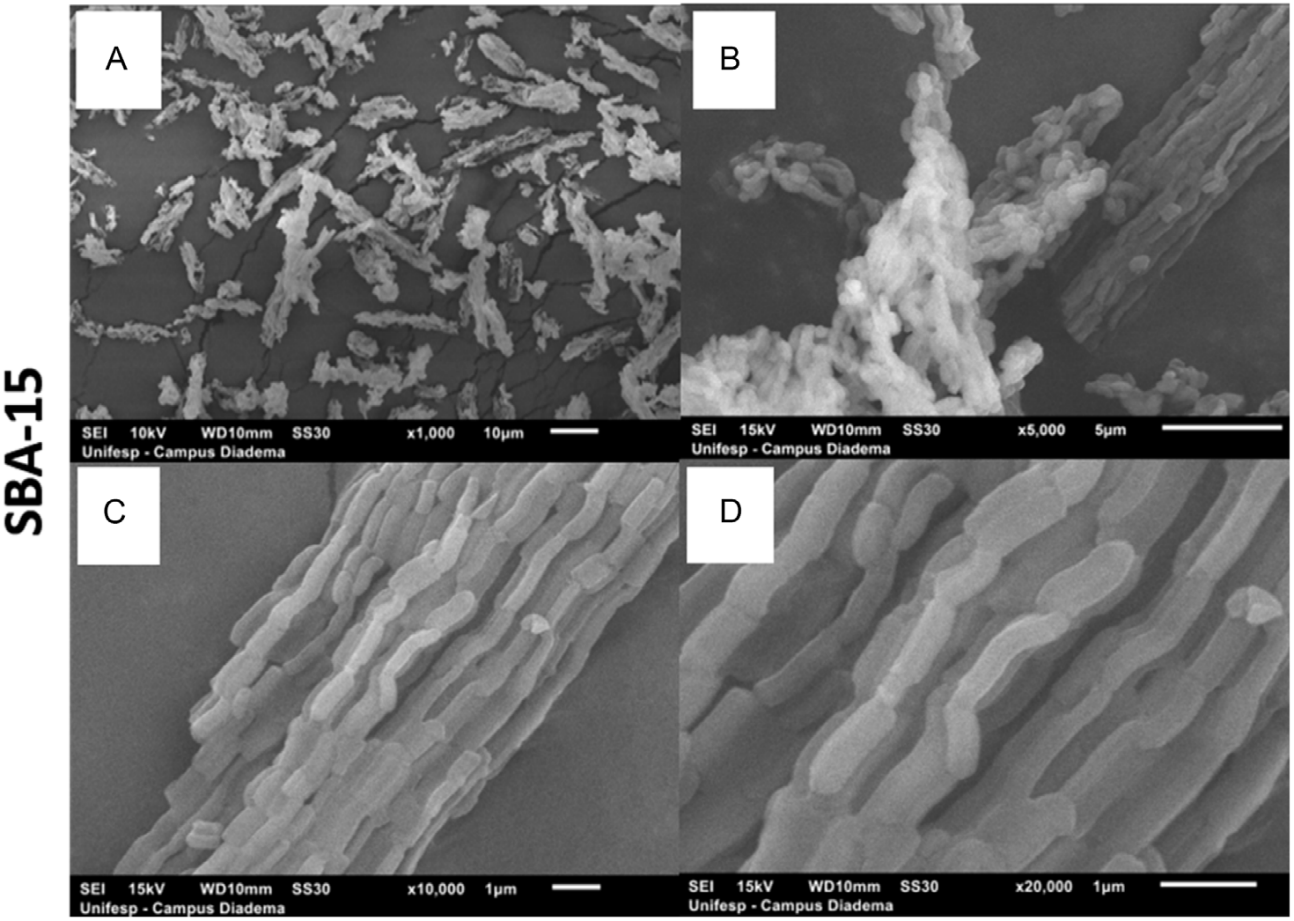
SBA‐15 SEM Micrographs in A) x1000, B) x5000, C) x10000, D) x20000, with particles in a rope‐like morphology.

**Figure 8:**
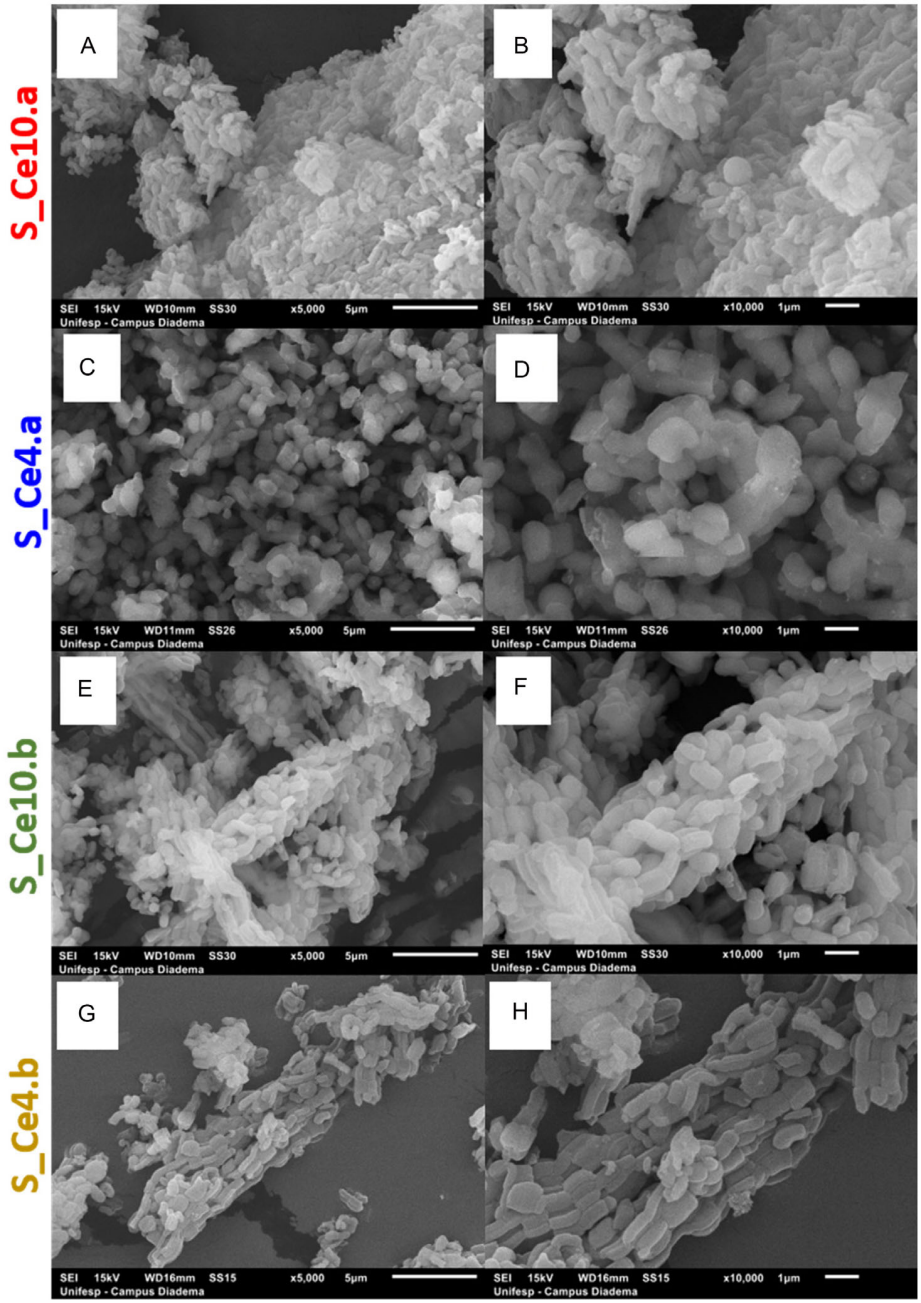
SEM micrographs for the nanocomposites in ×5000 and ×10000 of A,B) S_Ce10.a, C,D) S_Ce4.a, E,F) S_Ce10.b, and G,H) S_Ce4.b.

a) Specific surface area BET;

b) Pore diameter BJ;.

c) Pore volume;

d) Oxygen vacancy;

The SEM micrographs for the SBA‐15 and its nanocomposites are presented in **Figure** **7**A–D and **8**A–H, respectively. Pure SBA‐15 displays a characteristic morphology of interconnected rod‐like particles of ≈1 μm that are interconnected to form larger, wheat‐like aggregates, which is consistent with previous reports in the literature.^[^ ^82^, ^83^, ^84^ ^]^ For the postsynthesis nanocomposites, S_Ce4.b and S_Ce10.b, the SEM micrographs (Figure 8E–H) show no significant morphological changes, indicating that the bulk structure of SBA‐15 remains intact after CeO_2_ incorporation. In contrast, the nanocomposites prepared via direct synthesis appear more agglomerated, with smaller particle sizes observed for S_Ce10.a, and rounded shapes for S_Ce4.a (Figure 8A–D). These morphological differences are attributed to the stronger interactions between the reactants during the direct synthesis process.

Additionally, it is important to highlight both the benefits and limitations of particle size in practical CO_2_ capture systems. In large‐scale applications, particle size significantly impacts adsorption efficiency. While fine powders can enhance CO_2_ adsorption due to their high surface area, which can be tailored for improved performance, they also present challenges. In postcombustion capture systems, particularly those using fixed‐bed methods, smaller particles can lead to adsorption equilibrium limitations, rapid CO_2_ breakthrough, poor flowability, and high pressure drop, all of which can hinder their effectiveness under dynamic process conditions.^[^ ^85^, ^86^, ^87^ ^]^

**Figure 9:**
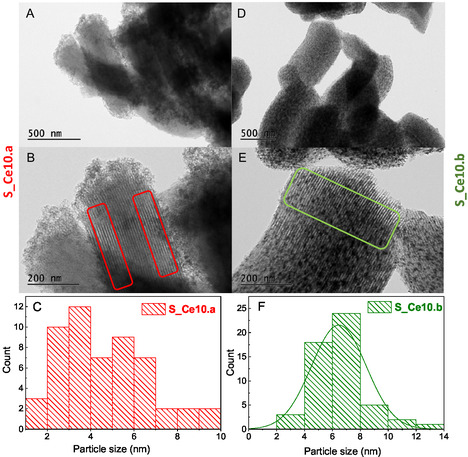
TEM images for the nanocomposite A,B) S_Ce10.a and D,E) S_Ce10.b. As well as the particle size distribution for C) S_Ce10.a and F) S_Ce10.b. The mesoporous channels and the well‐dispersed nanoparticles within them are highlighted by the rectangular markers.

**Figure** **9**A–F displays the TEM images for the nanocomposites S_Ce10.a and S_Ce10.b, along with the nanoparticle size distribution analyzed using ImageJ software. For both nanocomposites, the two‐bidimensional mesoporosity of SBA‐15 is clearly visible,^[^ ^88^ ^]^ with cerium nanoparticles uniformly dispersed throughout the silica framework, as indicated by the rectangular markers in Figure 9B,E. Furthermore, a slight variation in nanoparticle size is observed when comparing the PS and DS nanocomposites. The nanoparticle size for S_Ce10.a exhibits a polydisperse distribution, ranging from 2 to 10 nm, whereas S_Ce10.b shows a more uniform nanoparticle size distribution, primarily between 4 and 8 nm.

### 3.1 CO_2_ Adsorption Capacity

**Figure 10:**
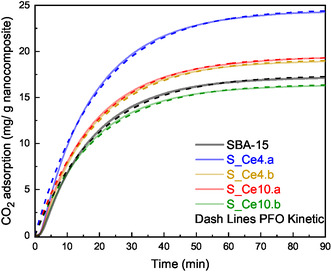
CO_2_ adsorption at 35 °C on SBA‐15 and SBA‐15:CeO_2_ nanocomposites, with pseudo‐first‐order kinetic fits (dashed line).

**Figure** **10** illustrates the CO_2_ adsorption capacity of all materials prepared in this study. Among them, S_Ce10.b was the only sample that exhibited lower adsorption compared to pure SBA‐15. When analyzing only the SBA‐15:CeO_2_ nanocomposites, the adsorption capacity, ranked from highest to lowest, follows the order: S_Ce4.a > S_Ce10.a > S_Ce4.b > S_Ce10.b. This result may correlate with the concentration of O.V., a critical property that significantly enhances the interaction between CO_2_ and metal oxide particles.^[^ ^52^, ^89^, ^90^ ^]^ Additionally, factors such as the amount of CeO_2_ incorporated into SBA‐15, pore diameter, pore volume, and specific surface area play crucial roles in determining the adsorption capacity.^[^ ^14^, ^35^, ^41^ ^]^ Moreover, a lower concentration of cerium oxide offers more adsorption sites compared to its higher‐concentration counterpart, as observed in the comparison of S_Ce4.a versus S_Ce10.a and S_Ce4.b versus S_Ce10.b.This phenomenon may be associated with a reduction in adsorption sites within SBA‐15, as larger quantities of nanoparticles tend to cover these sites, thereby impairing CO_2_ adsorption.

Table 5 summarizes the adsorption capacity of each nanocomposite at 35 °C, correlating these values with specific surface area, pore diameter, pore volume, and oxygen vacancy concentration. Although the sample S_Ce10.b exhibits a higher specific surface area compared to S_Ce10.a, the latter displays a larger pore size distribution and pore volume, suggesting a strong relationship between the mesoporous structure and CO_2_ adsorption. Furthermore, the samples containing 4% CeO_2_ show a greater specific surface area than those with 10%, further reinforcing the correlation between this property and CO_2_ adsorption.

### 3.2 CO_2_ Desorption

**Figure 11:**
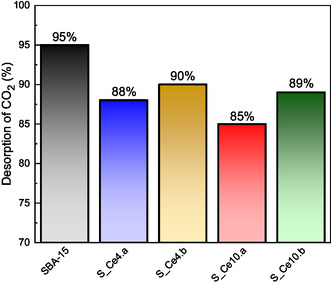
CO_2_ desorption at 35 °C from pure SBA‐15 and SBA‐15:CeO_2_ nanocomposites.

This study also aimed to examine the relationship between CO_2_ desorption and the structural properties of the nanocomposites. The percentage of CO_2_ desorbed for SBA‐15 and SBA‐15:CeO_2_ nanocomposites is presented in **Figure** **11**. The CO_2_ remaining in the samples after the desorption step requires higher energy to detach from the active sites due to stronger interactions with the nanocomposites. However, as demonstrated further, the CO_2_ is easily desorbed when the adsorption–desorption temperature is slightly higher, at 70 °C, indicating that the binding strength remains within the range of physisorption‐dominated interactions. Conversely, the CO_2_ that desorbs at lower temperatures is classified as weakly bound CO_2_, which can be easily removed following the adsorption step without significant energy input. This distinction highlights the role of oxygen vacancies in modulating CO_2_ adsorption strength, while still maintaining reversible adsorption behavior, characteristic of physisorption mechanisms.

As previously discussed, the relationship between higher concentrations of oxygen vacancies (O.V.) and CO_2_ adsorption is evident in these materials. Regarding the desorption process, oxygen vacancies also appear to play a crucial role. Samples S_Ce4.a and S_Ce10.a, which have the highest concentrations of O.V., exhibit stronger CO_2_ adsorption at their sites. The higher amount of cerium oxide in S_Ce10.a accounts for its greater proportion of strongly bound CO_2_ compared to S_Ce4.a. Comparing the postsynthesis materials, S_Ce4.b and S_Ce10.b show the same behavior, where the material with higher amount of CeO_2_ retains more CO_2_ adsorbed after the desorption process, although the values are practically the same. It is also important to note that all nanocomposites exhibit a greater number of strong adsorption sites than pure SBA‐15, indicating the influence of O.V. on the interaction with CO_2_.

These findings underscore the crucial influence of oxygen vacancies in determining the adsorption and desorption capacities of the materials. However, this study reveals that the quantity of strongly adsorbed CO_2_ is relatively low, positioning these nanocomposites as a promising choice for applications that prioritize weakly adsorbed CO_2_. Furthermore, the study presents the results of CO_2_ adsorption for the samples S_Ce4.a and S_Ce10.a over 20 cycles to assess the stability of these nanocomposites. In addition, the influence of temperature on the adsorption–desorption process was examined to better understand its impact on the material's performance.

### 3.3 CO_2_ Adsorption Kinetics

The CO_2_ adsorption kinetic model was analyzed to assess the adsorption characteristics of the nanocomposites. Several kinetic models are used to describe gas adsorption on solid sorbents, such as pseudo‐first‐order (PFO), pseudo‐second‐order (PSO), Elovich equation, film diffusion model, and Avrami model.^[^ ^91^, ^92^ ^]^ The PSO kinetics is normally used to describe chemical interactions, mainly binding gas to the surface by strong interactions. On the other hand, PFO is reported as well to describe kinetics of the gas adsorption on physical adsorbents, such as purely mesoporous silica materials. As demonstrated in our results, the materials tend to weak adsorption, achieving high desorption capacity of 97% and 95%, at 70 °C for the S_Ce4.a and S_Ce10.a samples, respectively. As the PFO kinetics model represents the reversible adsorption–desorption process, it was used to predict the CO_2_ adsorption behavior on the adsorbents studied in this article. In addition, PFO kinetic implies that the rate of adsorption is directly proportional to the number of available adsorption sites.^[^ ^18^, ^91^, ^92^, ^93^ ^]^ All samples exhibited a good correlation with the PFO kinetics.^[^ ^91^ ^]^ The equation for the PFO model, considering the initial conditions of *t* = 0, *q*_t_ = 0, and the condition at equilibrium *t* = ∞, *q*_t_ = *q*_e_, is presented below as Equation (1)

(1) $$q_{t}=q_{e}\left(1\;–\;e^{–kt}\right)$$

where *q*_*t*_ (mg g^−1^) represents the mass change due to CO_2_ adsorption at any given time *t*, while *q*_*e*_ (mg g^−1^) represents the CO_2_ adsorption at equilibrium. The rate constant of the PFO model is donated as *k*.

**Table** **6** reveals that the adsorption capacity of the samples prepared via the DS method is higher, suggesting a greater number of adsorption sites in these samples. Additionally, the rate constant (*k*) values are very similar across all samples, indicating comparable adsorption kinetics despite the observed differences in adsorption capacity.

**Table 6:** Adsorption kinetic parameters using PFO kinetic model for the test performed at 35 °C.

Samples	*q*_*exp*_ [mg g^−1^]	*k*_1_ [min^−1^]	*q*_*e*_ [mg g^−1^]	R^2^
SBA‐15	17.1	0.0518	17.4	0.994
S_Ce10.a	19.3	0.05334	19.4	0.997
S_Ce10.b	16.3	0.05391	16.5	0.996
S_Ce4.a	24.3	0.05193	24.6	0.996
S_Ce4.b	18.6	0.05283	19.2	0.994

Although the pseudo‐first‐order (PFO) model shows a strong correlation with our experimental data, it provides only a phenomenological description of the adsorption kinetics and does not fully capture the underlying adsorption mechanisms. To gain deeper insights into the rate‐limiting steps governing CO_2_ adsorption, further experimental studies could explore alternative kinetic models, such as the interparticle diffusion model and intraparticle diffusion model.^[^ ^91^, ^93^ ^]^ These models could help elucidate whether film diffusion, pore diffusion, or surface interactions play a dominant role in the adsorption process, thereby refining the understanding of CO_2_ uptake mechanism in SBA‐15:CeO_2_ composites.

### 3.4 Influence of Temperature

**Figure 12:**
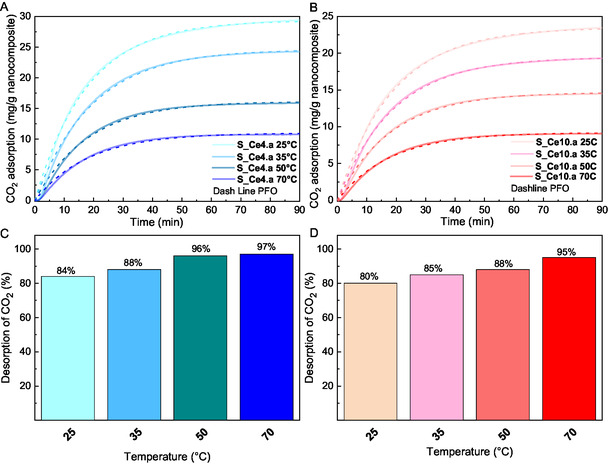
CO_2_ adsorption capacity and kinetics at 25 °C, 35 °C, 50 °C, and 70 °C for A) S_Ce4.a and B) S_Ce10.a, with corresponding CO_2_ desorption percentages for C) S_Ce4.a and D) S_Ce10.a.

To evaluate the influence of temperature on CO_2_ adsorption–desorption behavior, the nanocomposites S_Ce4.a and S_Ce10.a were tested at 25, 50 and 70 °C, in addition to 35 °C. These two samples were selected due to their higher CO_2_ adsorption capacity observed at 35 °C, with the aim of assessing whether desorption performance could be enhanced at slightly elevated temperatures, as they also exhibited lower desorption at 35 °C compared to the other samples. For all experiments, the temperature of adsorption and desorption was kept the same. **Figure** **12**A,B shows the experimental and theoretical adsorption capacity calculated by PFO kinetic. As expected, CO_2_ adsorption decreases with increasing temperature due to greater kinetic energy, which results in higher molecular mobility and, consequently, reduced adsorption capacity.^[^ ^94^ ^]^ For both materials, CO_2_ saturation was not achieved within 90 min of adsorption; however, the samples approached saturation more closely as the temperature increases. Overall, the S_Ce4.a (Figure 12A) continued to demonstrate a higher adsorption capacity than S_Ce10.a (Figure 12B) across all temperatures tested. Both samples adhere to PFO kinetics with strong correlation coefficients exceeding 0.99. The adsorption capacities and PFO kinetic parameters for the two materials are detailed in **Table** **7**.

**Table 7:** CO_2_ adsorption–desorption capacities and pseudo‐first‐order (PFO) kinetic parameters for samples S_Ce4.a and S_Ce10.a at 25, 35, 50, and 70 °C.

Samples	Temperature [°C]	CO_2_ ads [mg g^−1^]	CO_2_ des [%]	*k*_1_ [min^−1^]	*q*_*e*_ [mg g^−1^]	R^2^
S_Ce4.a	25	29.4	84	0.05189	29.4	0.994
	35	24.3	88	0.05334	24.6	0.997
	50	15.6	96	0.05391	16.1	0.996
	70	10.7	97	0.05193	11.0	0.996
S_Ce10.a	25	23.5	80	0.05306	23.5	0.996
	35	19.3	85	0.05334	19.5	0.997
	50	14.5	88	0.05522	14.7	0.996
	70	9.1	95	0.05614	9.2	0.995

Additionally, the CO_2_ desorption behavior was evaluated across different temperatures, as illustrated in Figure 12C,D. The results indicate that the amount of CO_2_ desorbed increases with rising temperature, approaching nearly 100% at 50 and 70 °C for sample S_Ce4.a (Figure 12C). A similar trend is observed for sample S_Ce10.a (Figure 12D); however, the higher concentration of nanoparticles enriched with oxygen vacancies enables this sample to retain greater amounts of CO_2_. While purely physiosorbed CO_2_ is expected to desorb completely at mild temperatures, the presence of oxygen vacancies in CeO_2_ can slightly enhance CO_2_ binding, leading to a fraction of CO_2_ requiring moderately higher temperatures for complete desorption. This observation corroborates previous discussions regarding the pivotal role of oxygen vacancies in enhancing CO_2_ interaction. Overall, these findings suggest that CO_2_ adsorption in these materials is primarily governed by physisorption mechanisms, as evidenced by the high desorption efficiency—97% for S_Ce4.a and 95% for S_Ce10.a at 70 °C.^[^ ^33^, ^95^, ^96^ ^]^

**Table** **8** presents a comparison of the CO_2_ adsorption capacity of the S_Ce.a nanocomposites studied in this work with various metal oxide‐based adsorbents, including CaO, MgO, and CeO_2_, as well as SBA‐15 impregnated with inorganic metal species. Given that the molar percentage of CeO_2_ impregnated in the mesoporous SBA‐15 structure is only 4% or 10%, the materials investigated in this study demonstrate promising adsorption performance compared to pure inorganic adsorbents, offering a balance between efficiency, cost‐effectiveness, and accessibility due to the relatively low amount of CeO_2_ required for impregnation.

**Table 8:** CO_2_ adsorption for materials reported in the literature, based on metal oxide (CeO_2_, MgO, CaO) or SBA‐15.

Adsorbent	Adsorption capacity [mg g^−1^]	Temperature [°C]	Reference
4% MgCeO_2_	26.4	30	Li et al.^[^ ^96^ ^]^
4% ZrCeO_2_	29.5	30	Li et al.^[^ ^96^ ^]^
4% LaCeO_2_	38.7	30	Li et al.^[^ ^96^ ^]^
4% CuCeO_2_	48.4	30	Li et al.^[^ ^96^ ^]^
CeO_2_	35.2	30	Li et al.^[^ ^96^ ^]^
CaO‐_coal fly ash_	7.8	30	Irshad et al.^[^ ^94^ ^]^
MgO‐_coal fly ash_	7.8	30	Irshad et al.^[^ ^94^ ^]^
CaOMgO‐_coal fly ash_	6.1	30	Irshad et al.^[^ ^94^ ^]^
CeO_2_‐2	2.6	50	Yoshikawa et al.^[^ ^104^ ^]^
CeO_2_‐3	5.8	50	Yoshikawa et al.^[^ ^104^ ^]^
CeO_2__dandelion‐like_	20.5	25	Zhao et al.^[^ ^105^ ^]^
CeO_2__comet‐like_	18.7	25	Zhao et al.^[^ ^105^ ^]^
CeO_2__bow‐tie‐like_	23.5	25	Zhao et al.^[^ ^105^ ^]^
SBA‐15‐ZrP	20.0	25	Zhang et al.^[^ ^106^ ^]^
SBA‐15	6.4	30	Mahendran et al.^[^ ^34^ ^]^
Mg‐SBA‐15‐C6	28.2	25	Zhao et al.^[^ ^107^ ^]^
Mg‐SBA‐15‐C3	29.5	25	Zhao et al.^[^ ^107^ ^]^
S_Ce10.a	19.3	35	This study
S_Ce10.a	23.5	25	This study
S_Ce4.a	24.3	35	This study
S_Ce4.a	29.4	25	This study

Additionally, this study highlights the high concentration of oxygen vacancies in SBA‐15:CeO_2_ composites, which not only enhance CO_2_ adsorption but also may improve catalytic activity when combined with species such as Ni and Cu. This dual functionality positions SBA‐15:CeO_2_ as a promising support material for applications requiring both adsorption and catalytic performance.^[^ ^97^, ^98^, ^99^, ^100^, ^101^, ^102^, ^103^ ^]^

### 3.5 Cyclability Test

**Figure 13:**
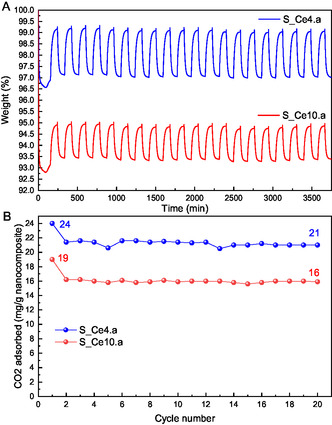
A) 20 cycles of CO_2_ adsorption capacity for the materials S_Ce4.a and S_Ce10.a, B) with respective values of CO_2_ adsorbed in mg per g of nanocomposite, for each cycle.

**Figure** **13**A,B illustrates the results of 20 cycles of CO_2_ adsorption–desorption tests conducted at 35 °C for samples S_Ce4.a and S_Ce10.a. In the first adsorption cycle, S_Ce4.a and S_Ce10.a exhibited CO_2_ uptake values of 24 and 19 mg g^−^^1^, respectively. Over subsequent cycles, these values decreased slightly to 21 and 16 mg g^−^^1^, indicating a minor reduction in adsorption capacity. This trend is consistent with previous CO_2_ adsorption–desorption analyses, where 12% of CO_2_ remained adsorbed in S_Ce4.a and 15% in S_Ce10.a after a single cycle at 35 °C. These results reflect the complete reversibility of weakly bound CO_2_, while a small fraction remains more strongly adsorbed, as discussed earlier. These findings suggest that both materials demonstrated effective stability, maintaining consistent CO_2_ adsorption performance across multiple cycles, which confirms their ability for efficient regeneration at low temperatures, with minimal CO_2_ retention postdesorption. Furthermore, considering the incorporation of catalytic species for CO_2_‐related reactions, SBA‐15:CeO_2_ nanocomposites emerge as a promising support. Their ease of adsorbent site regeneration ensures suitability for long‐term applications and highlights their potential in sustainable CO_2_ capture technologies.

## 4 Conclusion

In this study, we comprehensively investigated the structural, textural, and morphological properties of SBA‐15 and SBA‐15:CeO_2_ nanocomposites to understand their CO_2_ adsorption behavior. Our results emphasize the crucial role of oxygen vacancies in determining both adsorption and desorption capacities. The direct synthesis method provides materials with a higher concentration of oxygen vacancies, leading to enhanced CO_2_ interaction. Furthermore, a slightly stronger CO_2_ bond was observed in the DS samples, corroborating the relationship between ceria's structural defects and its interaction with CO_2_. Specifically, samples S_Ce4.a and S_Ce10.a exhibited superior performance with CO_2_ uptakes of 29.4 and 23.5 mg g^−^^1^ at 35 °C, compared to 17.1 mg g^−^^1^ for pristine SBA‐15, while retaining 12–15% of CO_2_ during desorption at the same temperature. Increasing the adsorption–desorption temperature to 70 °C reduced the retained CO_2_ to only 3–5%, confirming a predominantly physical interaction in the nanocomposites.

Additionally, these samples demonstrated stability over 20 adsorption–desorption cycles at 35 °C, although an initial decrease was observed after the first cycle due to incomplete CO_2_ desorption. Subsequent cycles showed consistent CO_2_ adsorption values, indicating their potential for extended use in cyclic processes. However, for this potential to be fully realized, practical challenges must be considered. Despite the improvement in the adsorption performance observed, we recognize that the application of fine power materials in practical CO_2_ capture systems, such as fixed or fluidized‐bed reactors, presents engineering challenges. Issues such as high pressure drop, poor flowability, and potential particle agglomeration are critical aspects that will need to be addressed in future optimization and scale‐up studies to enable their integration into industrial processes.

From this perspective in mind, while the observed adsorption capacity is modest relative to some materials reported in the literature, the insights provided regarding the formation of CeO_2_ rich in oxygen vacancies are highly valuable. They pave the way for the further development of catalysts specifically tailored for CO_2_ reforming. Moreover, the impregnation of 4% of CeO_2_ for S_Ce4.a sample preserves the possibility of incorporating additional species to enhance CO_2_ adsorption. Crucially, the direct synthesis route itself contributes to a more sustainable approach, offering advantages in scalability and potential cost‐effectiveness by streamlining the production process compared to more complex, multistep alternatives, such as postsynthesis methods. Overall, this research deepens our understanding of the interplay between structural properties and adsorption behavior in SBA‐15:CeO_2_ nanocomposites, offering critical insights for the optimization of materials based on CeO_2_ species tailored for CO_2_ capture applications.

## Conflict of Interest

The authors declare no conflict of interest.

## Supporting information

Supplementary Material

## Data Availability

The data that support the findings of this study are available from the corresponding author upon reasonable request.
