# Supplementary material for: Enhanced CO2 Adsorption on CeO2/SBA‐15: The Key Role of Oxygen Vacancies
Source: Chempluschem. 2025 Jul 16;90(9):e202500288. doi: 10.1002/cplu.202500288 (PMC12435142; doi:10.1002/cplu.202500288)
Supplement: Supplementary file 1 — Supplementary Material [file CPLU-90-e202500288-s001.pdf]

ChemPlusChem Supporting Information

Enhanced CO_2 Adsorption on CeO_2/SBA-15: The Key Role of Oxygen Vacancies

Danilo W. Losito,[a] Jessica A. F. Pedro,[b] Luís C. Cides-da-Silva,[b] Matheus C. R. Miranda,[a] Animesh Dutta,[c] Rafael M. Santos*,[c] Tereza S. Martins*,[a]

[a] D.W. Losito, Dr. M.C.R. Miranda, Dr. T.S. Martins
Department of Chemistry, Institute of Environmental, Chemical and Pharmaceutical Sciences, Federal University of Sao Paulo,
Rua São Nicolau 210 – Diadema, SP 09913-030, Brazil
E-mail: tsmartins@unifesp.br

[b] J.A.F. Pedro, Dr. L.C. Cides-da-Silva
Institute of Physics, Federal University of Sao Paulo,
Rua Do Matao 1371, Sao Paulo, SP 05508-090, Brazil

[c] Dr. A. Dutta, Dr. R.M. Santos
School of Engineering, University of Guelph,
50 Stone Rd E, Guelph, ON N1G 2W1, Canada
E-mail: santosr@uoguelph.ca

The supporting information contains additional data concerning the characterization of prepared materials as follows:

Figure S1 - Infrared spectrum.

Figure S2 - Thermogravimetric analysis.

Table S1 - SAXS peak area values 100.

Table S2 - Results of mass loss and T onset from thermogravimetric analysis.

Equation S1 - Scherrer's equation used to calculate the size of the crystallite.

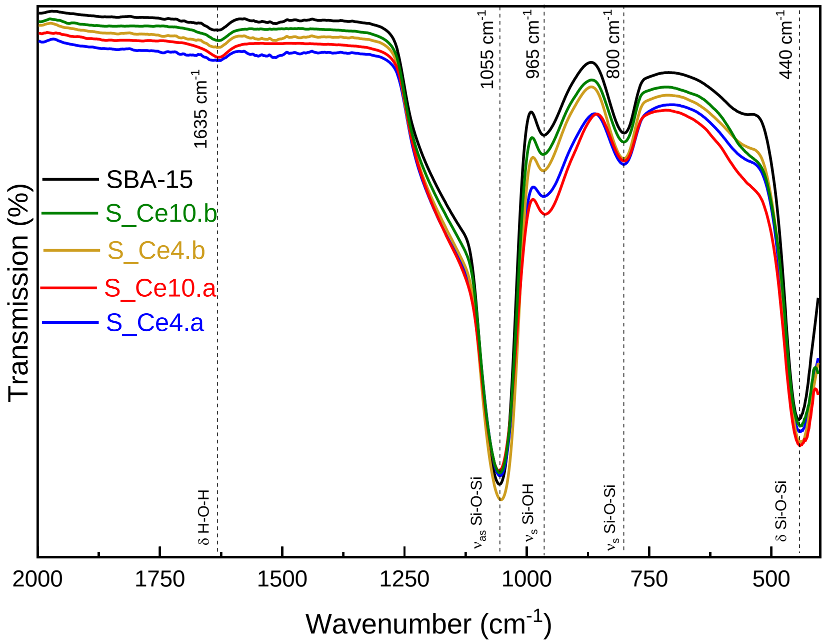

Figure S1. FTIR spectra for the SBA-15, S_Ce4a, S_Ce4b, S_Ce10a and S_Ce10b.

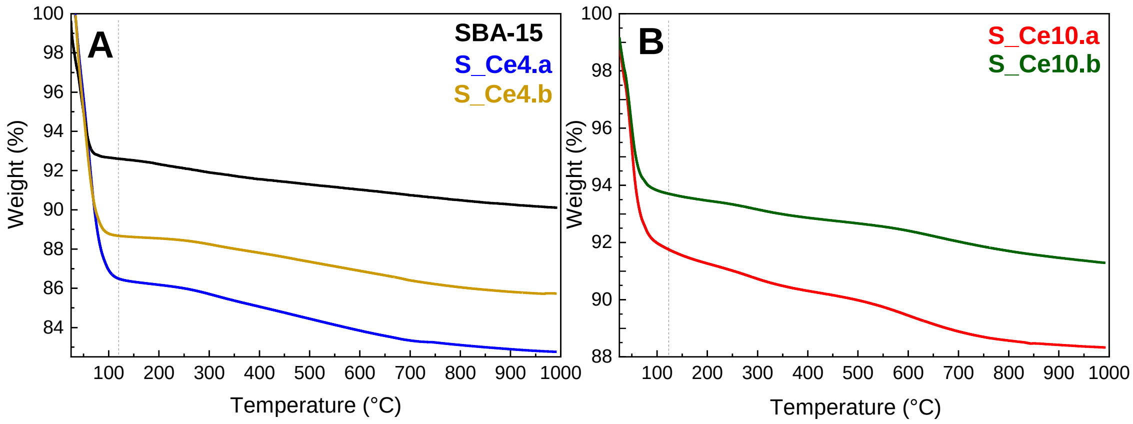

Figure S2. TGA results for the A) SBA-15, S_Ce4.a, S_Ce4.b, and B) S_Ce10.a and S_Ce10.b.

Table S1. Calculated values for the area under (100) peak of the SAXS curves.

Sample	Area under the peak (100)
SBA	0.019
S_Ce10.a	0.009
S_Ce4.a	0.018
S_Ce.b	0.004
S_Ce4.b	0.014

Table S2. Results obtained from the TG curves of SBA-15 and nanocomposites containing different amounts of CeO_2, prepared by post-synthesis and direct synthesis methods.

Samples	First event 25-120°C		Second event 120°C – 1000°C		
	[a]Δwt (%)	[b]T_{onset} (°C)	Δwt (%)	T_{onset} (°C)	[c]R(%)
SBA-15	7.4	25.4	2.5	210	90.1
S_Ce4.a	13.5	36.7	3.6	257	82.9
S_Ce4.b	11.2	36.9	2.9	299	85.9
S_Ce10.a	8.2	23.6	3.4	520	88.3
S_Ce10.b	6.3	23.5	2.6	670	93.2

[a] weight loss. [b] extrapolated onset temperature. [c] R = residue.

$$D_{hkl} = \frac{K\lambda}{\beta\cos(\theta)} \qquad \text{Equation (S1)}$$

$D_{(hkl)}$ = Crystallite size along the (hkl). K= shape factor; a dimensionless constant typically assumed to be 0.94 for spherical crystallites. λ= wavelength of X-ray radiation source (Cu Kα radiation, λ=0.15418 nm). β= Full width at half maximum (FWHM) of the (hkl) diffraction peak, expressed in radians. θ= Bragg angle of the (hkl) diffraction peak, expressed in radians.
